# Effects of liraglutide on diastolic function parameters in patients with type 2 diabetes and coronary artery disease: a randomized crossover study

**DOI:** 10.1186/s12933-020-01205-2

**Published:** 2021-01-07

**Authors:** Preman Kumarathurai, Ahmad Sajadieh, Christian Anholm, Ole P. Kristiansen, Steen B. Haugaard, Olav W. Nielsen

**Affiliations:** 1 Department of Cardiology, Copenhagen University Hospital of Bispebjerg, Bispebjerg Bakke 23, 2400 Copenhagen, Denmark; 2 Department of Internal Medicine, University Hospital of Amager, Copenhagen, Denmark; 3 Department of Nephrology and Endocrinolgy, University Hospital of Hillerød, Hillerød, Denmark; 4 Department of Endocrinology, University Hospital of Bispebjerg, Copenhagen, Denmark

**Keywords:** Diabetes, Diastolic function, Coronary artery disease, Echocardiography, Liraglutide, GLP-1 receptor agonists

## Abstract

**Background:**

Diastolic dysfunction is highly prevalent in patients with type 2 diabetes mellitus (T2DM) and is associated with overweight, glucose dysregulation and coronary artery disease (CAD). The GLP-1 receptor agonist, liraglutide, has shown to induce weight loss and improve metabolic factors, thus modulating factors associated with diastolic dysfunction. We have previously reported the effects of liraglutide on systolic function, and in this current study we explore the effects of liraglutide on diastolic function parameters in patients with stable CAD, preserved left ventricular ejection fraction (LVEF), and newly diagnosed T2DM.

**Methods:**

Thirty subjects were randomized to liraglutide or placebo intervention for 12 + 12-weeks in this double-blind cross-over study. 2D-echocardiography using tissue velocity imaging was used for assessment of diastolic function parameters. Early diastolic filling velocity (E), late atrial filling velocity (A), E-wave deceleration time (EDT) and E/A ratio was assessed from the pulse wave (PW)-Doppler velocity recording of the mitral inflow. Peak early diastolic annular velocities (e′) was measured from color tissue doppler images.

**Results:**

Liraglutide, when compared to placebo, induced a significant reduction in average e′ and lateral e′ velocities (– 0.57 cm/s [– 1.05 to − 0.08] and –0.74 cm/s [–1.32 to –0.15], respectively). Adjusted for the concomitant increase in HR (+ 6.16 bpm [0.79 to 11.54], the changes were not significant. No significant changes in other diastolic function parameters were observed.

**Conclusions:**

Liraglutide therapy did not improve any diastolic function parameters in subjects with T2DM, CAD, and preserved LVEF. Instead, a deterioration in e’ was observed, which was associated to an increase in heart rate induced by liraglutide therapy.

*Trial registration* Clinical Trial Registration: http://www.clinicaltrials.gov (unique identifier: NCT01595789) (first submitted May 8, 2012)

## Background

The prevalence of diastolic dysfunction in patients with type 2 diabetes mellitus (T2DM) is up to 75% and correlates with the degree of glucose dysregulation [1]. The condition is associated with factors such as overweight, hypertension, and coronary artery disease (CAD) [2]. Features of diastolic dysfunction such as impaired left ventricular (LV) relaxation, increased chamber stiffness, and increased LV filling pressure can be assessed non-invasively using 2D echocardiography [3]. Parameters of diastolic function derived from echocardiography have shown to predict mortality and hospitalization for heart failure [4, 5].

Glucagon-like peptide 1 (GLP-1) is an incretin hormone that reduces hyperglycemia, improves endothelial function, induce vasodilatory effects on arteries, and in clinical studies shown to reduce cardiovascular events, and improve cardiac function [6]. GLP-1 receptor agonists (RAs) mimic the effects of GLP-1 and is currently a well-established treatment option for T2DM [7]. GlP-1 RAs induce weight loss and improve glucometabolic factors [8], thus modulating risk factors associated with diastolic dysfunction. On this background, it is likely that treatment with GLP-1 RA would improve diastolic function.

In this randomized study, we examined the effect of the GLP-1 receptor agonist liraglutide on parameters of diastolic function in overweight patients with stable CAD, preserved left ventricular ejection fraction (LVEF), and newly diagnosed T2DM.

## Methods

We designed a randomized, double-blind, placebo-controlled 12 plus 12 weeks cross over study with a 2-week washout period. In- and exclusion criteria and the outline of the trial visits have been presented previously [9, 10]. In short, we included overweight patients with stable CAD, LVEF > 40% and newly diagnosed T2D within 24 months. Patients were recruited through patient files from selected hospitals in Copenhagen (Denmark) from May 2012 to October 2014. Patients on antidiabetic treatment before the study underwent a minimum 2-week washout period before the baseline visit. All subjects underwent 2D echocardiography, blood tests, anthropometric measurements, and blood pressure measurements at the beginning and end of each period (week 0, 12, 14, and 26). Subjects, investigators, and all caregivers were blinded to the treatment sequence. Data on systolic function has been published previously [10].

### Study drug

Patients who were on anti-diabetic treatment before the study underwent a minimum 2-week washout period before the first visit. Liraglutide or placebo were titrated in an identical manner in the active and placebo period, respectively: 0.6 mg liraglutide/placebo once daily (o.d.) was increased after 14 days to 1.2 mg o.d and to 1.8 mg o.d. after 28 days. All subjects had a backbone therapy of active metformin that were increased in a similar dose in both periods: 500 mg metformin twice daily was increased to 1000 mg + 500 mg after 14 days and to 1000 mg twice daily after 28 days.

### 2D echocardiography

The details of echocardiography protocol have been described previously [10]. In short, 2D echocardiography was performed at rest using a M5S transducer (Vivid E9, GE Vingmed Ultrasound, Horten, Norway). LVEF was calculated using the Simpson biplane method. Diastolic function parameters and their respective cut-off values were chosen as recommended in guideline [3]. Early diastolic filling velocity (E), late atrial filling velocity (A), E-wave deceleration time (EDT) and E/A ratio was assessed from the pulse wave (PW)-Doppler velocity recording of the mitral inflow. Peak early diastolic annular velocities (e′) was measured from color tissue doppler images with sample area placed in the septal, lateral, anterior and inferior parts of the mitral. Measurements was averaged from three beats. Furthermore, heart rate (HR) was measured from the ECG-recording from the echocardiography machine. Four investigators performed the echocardiography examinations (AS, OWN, OK, and PK). The off-line echocardiography analyses were performed by one observer (PK) who were blinded to the treatment protocol.

### Statistical analysis

No formal sample size calculation for diastolic function parameters was performed, as the study was primarily powered to detect changes in LVEF [10]. Continuous variables were summarized as the mean ± SD or medians with interquartile ranges, and categorical variables were summarized as percentages. The per-protocol population was defined as subjects who completed both measurement series in both intervention periods. Treatment effects of liraglutide and placebo were compared by using the paired t test. The association between variables of interest was examined by using linear regression. Chi- square test was used to compare differences in categorical variables between groups. The null hypothesis was rejected for values of P (two-sided) less than 0.05. All statistical analyses were performed with Stata 13.1 (StataCorp, College Station, Texas, USA).

## Results

In total, 41 subjects were randomly assigned to an intervention in this study. Screening, enrollment, and follow-up of the study population has been described previously [10]. Eleven subjects discontinued the study (2 subjects declined to participate after randomization, 9 subjects discontinued the study due to serious adverse events, intolerance to medication or other reasons). Thus, 30 subjects were available for the per protocol analyses. See Additional file 1: Figure S1.

The baseline characteristics of the per-protocol study population is shown in Table 1. The study population consisted of 80% men and had an average age of 63 years. Mean weight was 98.6 kg corresponding to a mean BMI of 32 kg/m^2^. Pre-study anti-diabetic therapy and cardiovascular medication is shown in Table 1.

**Table 1:** Baseline characteristics of the study population

Characteristics	Total (n = 30)
Clinical characteristics	
Age, year	63.1 (6.6)
Male Sex, n (%)	24 (80)
Weight, kg	98.6 (18.0)
BMI, kg/m^2^	32.0 (5.2)
Waist, cm	112.2 (10.9)
Systolic blood pressure, mmHg	142.7 (19.6)
Diastolic blood pressure, mmHg	79.7 (10.0)
Heart rate, bpm	68.6 (10.1)
Risk factors	
Smoker, n (%)	14 (36)
Hypertension, n (%)	23 (77)
Coronary artery disease	
Previous MI, n (%)	16 (53)
Previous CABG, n (%)	13 (43)
Previous PCI, n (%)	16 (53)
Coronary stenosis, medical therapy only, n (%)	2 (7)
Biochemistry	
Fasting blood glucose, mmol/l	6.4 (1.4)
HbA1C, %	6.3 (0.5)
LDL-cholesterol, mmol/l	2.3 (0.7)
eGFR, ml/min	78.3 (11.5)
Medication	
Beta blockers, n (%)	20 (67)
Calcium antagonists, n (%)	15 (50)
ACE-I, ARB, n (%)	20 (67)
Statins, n (%)	29 (97)
Ivabradine, n (%)	1 (3)
Diuretics, n (%)	10 (33)
Aspirin, n (%)	29 (97)
Pre-study diabetes medication	
Biguanide (metformin), n (%)	12 (40)
Sulfonylurea, n (%)	1 (3.3)
Diet and lifestyle therapy only, n (%)	18 (60)

Data are expressed as the mean (SD) or n (%)

*ACE-I* angiotensin converting enzyme inhibitor, *ARB* angiotensin receptor blocker, *BMI* body mass index, *bpm* beats per minute, *CABG* coronary artery bypass grafting, *HbA1C* glycated hemoglobin, *MI* myocardial infarction, *PCI* percutaneous coronary intervention, *LDL* low-density lipoprotein, *eGFR* estimated glomerular filtration rate

Table 2 shows the baseline left ventricular diastolic parameters for the per-protocol population. Signs of diastolic dysfunction occurred in the majority of the subjects: 96.7% had a reduced eʹ, 30% had an enlarged left atrium, and 46.7% had an average E/eʹ value above 14. However, subjects were well-compensated because the majority had a TR-velocity ≤ 2.8 m/s indicating a normal pulmonary systolic pressure. Systolic function parameters have been described previously [10]. The mean LVEF at baseline was 58.9%. Average sʹ velocity was 6 cm/sec and global longitudinel strain was 16.4%.

**Table 2:** Baseline diastolic function parameters

Parameters	Total (n = 30)
LVMI, gram/m^2^	83.4 (23.3)
RWT, cm	0.32 (0.1)
Left atrial volume index, ml/m^2^	29.3 (8.3)
E/A ratio	0.95 (0.1)
Peak E velocity, m/s	0.8 (0.2)
Deceleration time, ms	239.22 (42.3)
Average E/eʹ	15.36 (6.4)
Lateral eʹ velocity, cm/s	6.59 (2.4)
Septal eʹ velocity, cm/s	5.1 (1.4)*
Peak TR velocity, m/s	2.2 (0.5)**
Average eʹ, cm/s	5.7 (1.6)
Systolic function	
LVEF, baseline, %	58.9 (7.2)
Average s’ velocity, cm/s	6.0 (1.3)
GLS, %	16.4 (2.9)
Number of subjects with signs of diastolic dysfunction	
Average E/eʹ > 14, n (%)	14 (46.7%)
Lateral eʹ velocity < 10 cm/s or septal eʹ velocity < 7 cm/s, n (%)	29 (96.7%)
Peak TR velocity > 2.8 m/s	2 (7.4%)**
Left atrial volume index > 34 ml/m^2^	9 (30%)

Data are expressed as mean (SD) or n (%)

*LVMI* left ventricular mass index, *LVEF* left ventricular ejection fraction, *RWT* relative wall thickness

^*^ n = 29; **n = 27

Diastolic function parameters before and after intervention are shown in Table 3. A significant reduction in average e’ and lateral e’ velocities was observed after 12 weeks of liraglutide treatment as compared with placebo (– 0.57 cm/s; p = 0.023) and – 0.74 cm/s; p = 0.016 respectively).

**Table 3:** Changes in diastolic function parameters

	Before liraglutide	After liraglutide	Before placebo	After placebo	N for treatment effect	Treatment effect for liraglutide	Treatment effect for placebo	**Difference (95% CI)**	**p-value**
Diastolic function parameters									
Average E/eʹ	14.53 (5.76)	14.23 (5.55)	15.28 (6.21)	14.5 (5.98)	29	− 0.20 (2.61)	− 0.85 (3.44)	0.65 (− 0.86 to 2.16)	0.386
Lateral eʹ velocity, cm/s	7.02 (2.15)	6.62 (2.11)	6.52 (2.62)	6.85 (1.98)	30	− 0.40 (1.29)	0.33 (1.55)	− 0.74 (− 1.32 to − 0.15)	0.016
Septal eʹ velocity, cm/s	5.34 (1.32)	5.01 (1.37)	5.24 (1.52)	5.3 (1.44)	29	− 0.31 (0.94)	− 0.03 (1.05)	− 0.28 (− 0.85 to 0.29)	0.326
Average eʹ, cm/s	6.11 (1.55)	5.62 (1.47)	5.76 (1.72)	5.84 (1.54)	30	− 0.49 (0.96)	0.08 (0.95)	− 0.57 (− 1.05 to − 0.08)	0.023
Peak TR velocity, m/s	2.2 (0.55)	2.09 (0.6)	2.23 (0.52)	2.17 (0.53)	26	− 0.06 (0.49)	− 0.09 (0.27)	0.03 (− 0.15 to 0.2)	0.732
Left atrial volume index, ml/m^2^	30.36 (9.04)	26.42 (9.34)	29.78 (8.14)	26.77 (8.88)	30	− 3.95 (5.59)	− 3.01 (7.9)	− 0.94 (− 4.76 to 2.89)	0.621
Other measures of diastolic function and heart rate									
E/A ratio	1.06 (0.57)	0.89 (0.22)	0.94 (0.25)	1.02 (0.51)	29	− 0.18 (0.51)	0.08 (0.33)	− 0.26 (− 0.55 to 0.03)	0.082
Mitral valve peak E velocity, cm/s	0.83 (0.19)	0.75 (0.16)	0.8 (0.16)	0.78 (0.17)	29	− 0.08 (0.13)	− 0.02 (0.12)	− 0.06 (− 0.13 to 0.02)	0.142
Deceleration time, milliseconds	242.55 (49.71)	234.03 (50.97)	247.95 (48.19)	246.83 (61.02)	29	− 9.90 (51.06)	0.07 (58.8)	− 9.96 (− 39.78 to 19.85)	0.499
Heart rate, bpm	67.67 (12.59)	74.28 (13.08)	68.03 (11.56)	68.48 (9.25)	30	6.61 (10.63)	0.45 (8.12)	6.16 (0.79 to 11.54)	0.026

Data are expressed as the mean (SD)

No significant changes in other diastolic function parameters were observed: average E/eʹ, E/A ratio, Peak E velocity, Peak TR velocity, left atrial volume, deceleration time, septal eʹ velocity.

A significant increase in baseline HR after liraglutide therapy was observed (+ 6.16 bpm [95% CI 0.79 to 11.54]).

Changes in resting blood pressure and metabolic variables are shown in Additional file 2: Table S1.

### Regression analyses

Liraglutide treatment was significantly associated with the changes in average e’ (beta-coefficient − 0.57; p = 0.025), but this correlation disappeared when adjusted for the concomitant increase in HR (beta-coefficient − 0.48; p = 0.071). Similarly, no significant associations between lateral and septal e’ and liraglutide treatment were found before or after adjustment for HR (Table 4).

**Table 4:** Linear regression models showing the association between liraglutide treatment and changes in diastolic parameters

	β-coefficient	95% CI	p-value
Dependent variable			
Changes in average eʹ			
Univariate	− 0.57	− 1.06 to − 0.07	0.025
Adjusted for changes in HR	− 0.48	− 0.995 to 0.043	0.071
Changes in lateral eʹ			
Univariate	− 0.74	− 1.47 to 0.002	0.051
Adjusted for changes in HR	− 0.73	− 1.51 to 0.06	0.068
Changes in septal eʹ			
Univariate	− 0.28	− 0.80 to 0.24	0.289
Adjusted for changes in HR	− 0.13	− 0.67 to 0.41	0.630

Independent variable: treatment (liraglutide)

*HR* heart rate

We did not find any association between changes in average eʹ, lateral eʹ, septal eʹ, E/eʹ ratio and changes in HR, weight, HbA1C, systolic BP and diastolic BP during liraglutide period. During placebo period we only found an association between changes in average E/eʹ and changes in HbA1C (Table 5).

**Table 5:** Regression coefficients (β), R-squared values and p-values from linear regression analysis

Dependent variable	Independent variable	Placebo			Liraglutide		
		β	R^2^	p-value	β	R^2^	p-value
ΔAverage eʹ	Δheart rate	− 0.012	0.010	0.602	− 0.016	0.033	0.339
ΔAverage eʹ	Δweight	0.054	0.022	0.437	< 0.001	< 0.001	0.993
ΔAverage eʹ	ΔHbA1C	− 0.010	< 0.001	0.981	0.147	0.003	0.783
ΔAverage eʹ	ΔSystolic BP	0.006	0.010	0.603	0.002	0.001	0.882
ΔAverage eʹ	ΔDiastolic BP	− 0.010	0.008	0.639	− 0.023	0.081	0.127
Lateral eʹ velocity	Δheart rate	< 0.001	< 0.001	0.993	− 0.002	< 0.001	0.930
Lateral eʹ velocity	Δweight	0.008	< 0.001	0.944	− 0.040	0.012	0.572
Lateral eʹ velocity	ΔHbA1C	− 0.002	< 0.001	0.997	0.126	0.001	0.861
Lateral eʹ velocity	ΔSystolic BP	0.014	0.020	0.459	− 0.014	0.036	0.319
Lateral eʹ velocity	ΔDiastolic BP	− 0.016	0.009	0.628	− 0.026	0.061	0.189
Septal eʹ velocity	Δheart rate	− 0.029	0.044	0.277	− 0.023	0.070	0.167
Septal eʹ velocity	Δweight	0.111	0.074	0.154	0.021	0.006	0.685
Septal eʹ velocity	ΔHbA1C	0.084	0.001	0.857	0.452	0.028	0.388
Septal eʹ velocity	ΔSystolic BP	0.000	< 0.001	0.983	− 0.007	0.018	0.489
Septal eʹ velocity	ΔDiastolic BP	− 0.015	0.017	0.502	− 0.025	0.107	0.084
Average E/eʹ	Δheart rate	− 0.063	0.023	0.423	− 0.061	0.054	0.225
Average E/eʹ	Δweight	0.118	0.008	0.631	0.241	0.107	0.083
Average E/eʹ	ΔHbA1C	2.889	0.135	0.046	− 1.072	0.020	0.461
Average E/eʹ	ΔSystolic BP	− 0.008	0.002	0.836	− 0.010	0.004	0.735
Average E/eʹ	ΔDiastolic BP	− 0.045	0.014	0.539	0.055	0.064	0.185

*HbA1C* glycated hemoglobin, *BP* blood pressure

## Discussion

In this double-blind, placebo-controlled study, we examined the effect of 12 weeks of liraglutide treatment on diastolic function parameters as assessed by 2D echocardiography. We demonstrated that liraglutide did not improve any diastolic parameters. Instead, we observed a reduction in eʹ suggesting a mild worsening of diastolic function, that may be attributed to an increase in HR induced by liraglutide therapy.

Obesity and T2DM are associated with diastolic dysfunction [11]. Accordingly, weight loss and improvement in metabolic factors have shown to improve parameters of diastolic function [12]. The baseline data in our population revealed a high proportion of subjects that displayed one or more abnormal diastolic function parameters. However, despite significant weight loss and improvement in glucometabolic parameters during the liraglutide period, we did not find any improvement in diastolic function parameters. Instead, eʹ, a commonly used marker for diastolic function deteriorated. It is not clear if this seemingly worse eʹ represents a true worsening of diastolic function or is a result of the increased heart rate secondary to liraglutide. We observed a significant negative association between liraglutide treatment and average eʹ in our regression analyses. Furthermore, when adjusting for the HR changes, the association between eʹ and liraglutide became non-significant suggesting that the increase in resting HR during liraglutide therapy led to a reduced e’.

Previous studies have shown the inverse relation between HR and diastolic function. Esfandiari et al. showed in a small cohort of subjects with and without HF that atrial pacing impaired diastolic function parameters [13]. Burns et al. showed in 15 subjects that accelerated HR by atrial pacing, induced a significant decrease in e’ [14]. However, we did not find any direct correlation between changes in HR and changes in average eʹ in our study. Nevertheless, the HR increase associated with liraglutide has been of concern, namely in subjects with heart failure with reduced ejection fraction [15–17]. An increase in death or rehospitalization for heart failure was observed in subjects with T2DM and heart failure with reduced ejection fraction [16]. Whether the adverse events were due to any detrimental effect of liraglutide on myocardial relaxation or due to HR increase was not assessed in that study.

Effect of GLP-1 RA on diastolic function in previous trials are in line with our present findings. A randomized, placebo-controlled study of 33 patients with T2DM patients showed that liraglutide blunted the effect of supervised training on diastolic function (eʹ), which in the placebo groups improved [18]. In a randomized open-label study, patients with T2DM and subclinical heart failure did not improve diastolic function parameters after 18 weeks of liraglutide therapy when compared to glimepiride treatment [19]. In a small study of 23 patients LV filling pressure was improved after liraglutide therapy, but no effect on myocardial relaxation (eʹ) was seen [20]. Exenatide therapy did not improve either exercise capacity or diastolic function in a randomized, placebo-controlled study [21]. The LIVE study examined the effect of liraglutide on LV function in subjects with LVEF ≤ 40% [15]. A small improvement in E/eʹ was indeed observed; however, the authors note that the clinical relevance of these findings was unclear [15, 22]. In a prospective, observational, non-randomized study, 6 months of liraglutide therapy improved several diastolic function parameters [23]. However, the study was not randomized or blinded and did not have a prober control-group. In a systemic review and network metanalysis no clear signal on e’ was found in GLP-1 RA studies [24].

Potential strength and limitations of the study should be mentioned. The use of crossover design reduced the within-patient variation thus granting appropriate power in our study to address the primary endpoints. We did not perform a power calculation with diastolic function parameters as the study was primarily powered to detect changes in LVEF [10]. Furthermore, carry over effects were not assessed. Thus, the data presented here are exploratory analyses and should be regarded as hypothesis-generating. We analyzed only echocardiographic measures of diastolic dysfunction. Thus, any improvement in parameters associated with diastolic dysfunction, such as dyspnea or natriuretic peptides, were not evaluated in this study. The homogeneity of the cohort limits the application of our results to other patient groups such as patients with severe heart failure, patients with long-standing diabetes with poor glycemic control, or patients with severe CAD.

We previously demonstrated that liraglutide did not improve systolic function [10]. Our current findings suggest that liraglutide does not improve diastolic function either. Instead, we observed deterioration of one of the parameters of diastolic function. As previously described, 48-h HR monitoring revealed an increase in HR of 8.1 bpm after liraglutide therapy when compared to placebo [25]. Although, the resting HR during echocardiography described here were of smaller magnitude, our analyses suggest that the increase in HR may explain this deterioration in diastolic function. If so, HR-limiting medications, such as beta-blockers or sinus node inhibitors, in combination with GLP-1 RAs may improve or at least reduce deterioration of diastolic function parameters. Despite our findings suggesting deterioration of diastolic function, it was demonstrated in the LEADER study [7], that liraglutide had an overall beneficial effect on cardiovascular outcome. This may be due to the pleiotropic effect of liraglutide and GLP-1 RAs in general [26].

## Conclusions

In conclusion, our study showed that liraglutide therapy do not improve any diastolic function parameters in subjects with T2DM, CAD, and preserved LVEF. Instead, we observed a deterioration in e’ that may be mediated by the HR increase induced by liraglutide therapy.

## Supplementary Information

**Additional file 1: Figure S1.** Screening, enrollment, and follow-up of the study population.

**Additional file 2: Table S1.** Effect of liraglutide versus placebo on blood pressure and metabolic variables. **Table S2.** Regression coefficients (β), R-squared values and p-values from linear regression analysis.

## Data Availability

The data that support the findings of this study are available from the corresponding author upon reasonable request.
