# Supplementary material for: Effects of liraglutide on diastolic function parameters in patients with type 2 diabetes and coronary artery disease: a randomized crossover study
Source: Cardiovasc Diabetol. 2021 Jan 7;20:12. doi: 10.1186/s12933-020-01205-2 (PMC7791686; doi:10.1186/s12933-020-01205-2)
Supplement: Supplementary file 2 — Additional file 2: Table S1. Effect of liraglutide versus placebo on blood pressure and metabolic variables. Table S2. Regression coefficients (β), R-squared values and p-values from linear regression analysis. [file 12933_2020_1205_MOESM2_ESM.docx]

**Table S1. Effect of liraglutide versus placebo on blood pressure and metabolic variables**

|  | **Treatment effect** | | **Difference** | **95% CI** | **p-value** |
| --- | --- | --- | --- | --- | --- |
|  | **Liraglutide** | **Placebo** |  |  |  |
| Weight, kg | -4.17 (3.49) | -0.98 (2.62) | -3.18 (4.31) | -4.79 to -1.57 | <0.001 |
| Systolic blood pressure, mmHg | -8.10 (17.27) | -3.17 (16.07) | -4.93 (23.68) | -13.78 to 3.91 | 0.263 |
| Diastolic blood pressure, mmHg | -3.13 (12.11) | -3.83 (8.75) | 0.70 (17.06) | -5.67 to 7.07 | 0.826 |
| HbA1c, mmol/mol | -4.70 (3.98) | -0.60 (4.90) | -4.10 (6.02) | ‑6.35 to ‑1.85 | <0.001 |
| LDL-cholesterol, mmol/L | -0.25 (0.72) | -0.17 (0.63) | -0.08 (0.96) | -0.47 to 0.30 | 0.657 |
| Fasting plasma insulin, pmol/L | -11.73 (57.54) | -1.69 (43.79) | -10.04 (68.27) | -36.01 to 15.93 | 0.469 |
| Fasting blood glucose, mmol/L | -0.99 (1.11) | -0.62 (0.96) | -0.36 (1.06) | -0.76 to 0.03 | 0.125 |
|  |  |  |  |  |  |

Data are expressed as the mean (SD). BMI, body mass index; HbA1c, glycated hemoglobin; LDL, low-density lipoprotein; HOMA IR, homeostasis model analysis of insulin resistance.

**Table S2.** Regression coefficients (β), R-squared values and p-values from linear regression analysis.

|  |  | Placebo | | | | |  | | Liraglutide | | | | | |
| --- | --- | --- | --- | --- | --- | --- | --- | --- | --- | --- | --- | --- | --- | --- |
| **Dependent variabel** | **Independent variabel** | **β** | **R^2^** | **p-value** | |  | | **β** | | **R^2^** | | **p-value** | |  |
|  |  |  |  | |  | |  | |  | |  | |  | |
| ΔHR | ΔResting systolic blood pressure | 0.070 | 0.024 | | 0.440 | |  | | -0.024 | | 0.002 | | 0.812 | |
| ΔHR | ΔResting diastolic blood pressure | 0.410 | 0.254 | | 0.007 | |  | | 0.130 | | 0.031 | | 0.377 | |
| ΔHR | ΔWeight | -0.713 | 0.076 | | 0.165 | |  | | -0.089 | | 0.001 | | 0.852 | |
| ΔHR | ΔHbA1C | -2.848 | 0.032 | | 0.373 | |  | | 3.670 | | 0.022 | | 0.465 | |
| ΔHR | ΔFasting blood glucose | -1.026 | 0.025 | | 0.434 | |  | | 0.662 | | 0.009 | | 0.645 | |
|  |  |  |  | |  | |  | |  | |  | |  | |
|  |  |  |  | |  | |  | |  | |  | |  | |
|  |  |  |  | |  | |  | |  | |  | |  | |
|  |  |  |  | |  | |  | |  | |  | |  | |
|  |  |  |  | |  | |  | |  | |  | |  | |
|  |  |  |  | |  | |  | |  | |  | |  | |
|  |  |  |  | |  | |  | |  | |  | |  | |
|  |  |  |  | |  | |  | |  | |  | |  | |
|  |  |  |  | |  | |  | |  | |  | |  | |

HbA1C, glycated hemoglobin; HR, heart rate; SDNN, standard deviation of all normal RR-intervals; HF, high frequency power; LF, low frequency power; TP, total power
